# Uma Causa Rara de Hipoxemia após Cirurgia Ortopédica num Doente Idoso

**DOI:** 10.36660/abc.20210409

**Published:** 2022-03-10

**Authors:** Pedro Carvalho, Daniela Meireles, José Luís Martins, Marco Costa, Ana Briosa Neves

**Affiliations:** 1 Centro Hospitalar do Baixo Vouga Cardiology Department Aveiro Portugual Centro Hospitalar do Baixo Vouga – Cardiology Department, Aveiro – Portugual; 2 Centro Hospitalar do Baixo Vouga Internal Medicine Department Aveiro Portugal Centro Hospitalar do Baixo Vouga – Internal Medicine Department,Aveiro – Portugal; 3 Centro Hospitalar Universitário de Coimbra Cardiology Department Coimbra Portugal Centro Hospitalar Universitário de Coimbra – Cardiology Department, Coimbra – Portugal

**Keywords:** Forame Oval Patente, Hipóxia, Dispositivos de Oclusão Vascular

## Introdução

Várias doenças podem causar hipoxemia pós-operatória, especialmente em pacientes idosos. Entretanto, um novo shunt cardíaco é uma complicação muito rara e inesperada nesse cenário. Este estudo relata um caso de hipoxemia refratária após cirurgia ortopédica devido a um shunt da direita para a esquerda via um forame oval patente (FOP).

## Relato de caso

Um paciente do sexo masculino de 71 anos foi submetido a cirurgia eletiva para artroplastia do quadril esquerdo, com anestesia locorregional. Seu histórico médico incluía obesidade, hipertensão, diabetes mellitus e um acidente vascular cerebral. Ele não tinha histórico de doença cardiopulmonar.

**Figura 1:**
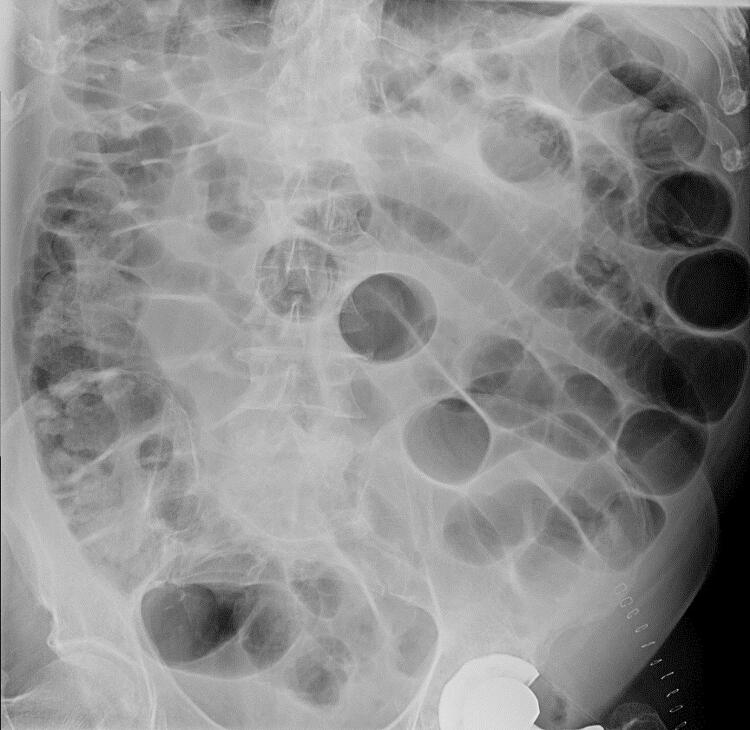
Radiografia abdominal demonstrando distensão abdominal.

O primeiro dia do pós-operatório foi complicado por obstrução intestinal ( Figura 1 ). A alimentação foi reiniciada quatro dias depois, mas a distensão abdominal e a redução dos movimentos peristálticos persistiram. No 15º dia de pós-operatório, o paciente apresentou hipoxemia refratária grave, com uma saturação de O_2_ (O_2_sat) de 75%, chegando apenas a 86% com oxigenoterapia de alto fluxo (F_i_O_2_ 90-100%). Apesar disso, o paciente estava calmo, sem apresentar sinais de desconforto respiratório. A pressão arterial era 110/75 mmHg, a frequência cardíaca era 76 bpm e a temperatura era 36 ºC. A auscultação cardíaca e a pulmonar estavam normais. Não havia distensão venosa jugular, edema periférico ou cianose.

A gasometria arterial confirmou hipoxemia grave, com uma pO_2_ de 38 mmHg em F_i_O_2_ de 28%, chegando apenas a 49 mmHg em F_i_O_2_ 100%. O hemograma estava normal, exceto pelo alto nível de dímeros-D (1608 ng/mL). O eletrocardiograma e o ecocardiograma à beira do leito estavam normais. O paciente foi submetido a uma angiotomografia computadorizada arterial de tórax que não revelou sinais de embolia pulmonar ou doença pulmonar parenquimatosa significativa. Nos dias seguintes, a O_2_sat foi mantida em 85-89% apesar da cânula nasal de alto fluxo de oxigênio, independentemente de o paciente estar em pé, em posição supina ou em decúbito lateral esquerdo.

**Figura 2:**
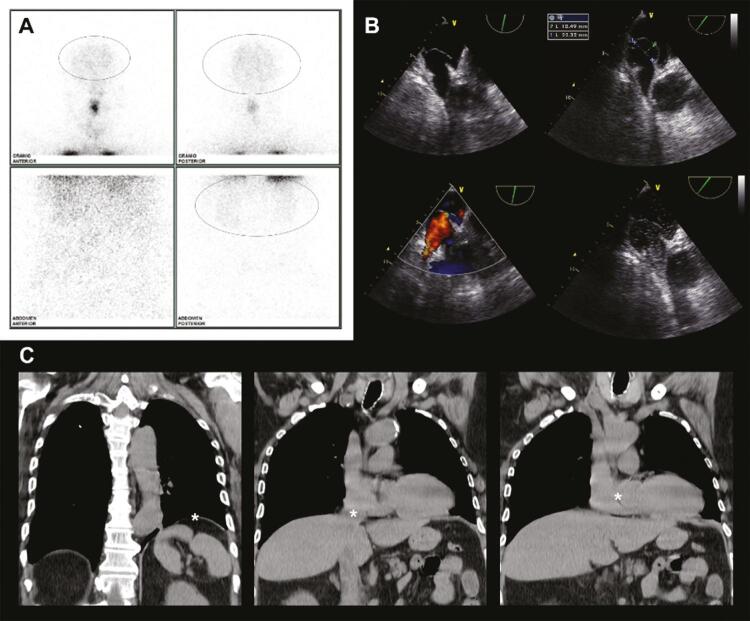
Painel A: Cintilografia de V/Q demonstrando captação, pelo cérebro e pelo rim, de macroagregado de albumina 99m Tc; Painel B: ETE demonstrando um aneurisma do septo interatrial e FOP, com um grande shunt D-E em repouso, visível por Doppler colorido e injeção de solução salina agitada; Painel C: Imagens de TC demonstrando a elevação do hemidiafragma esquerdo* alterando o eixo da veia cava inferior supra-hepática* e a posição do coração* horizontalmente.

Foi realizada uma cintilografia de ventilação e perfusão pulmonar (V/Q), demonstrando a ausência de desequilíbrio V/Q, mas revelando captação do marcador pelo cérebro e pelo rim, sugerindo shunt D-E ( Figura 2A ). O ecocardiograma transesofágico (ETE) revelou um aneurisma do septo interatrial e um FOP com um grande shunt D-E em repouso, visível por Doppler colorido e injeção de solução salina agitada ( Figura 2B ). Nas análises de imagens de tomografia computadorizada (TC), verificou-se que a distensão intestinal havia causado a elevação do hemidiafragma esquerdo, alterando o eixo da veia cava inferior supra-hepática e a posição do coração (e, consequentemente, a posição do septo interatrial) horizontalmente ( Figura 2C ). Em imagem de contraste, observou-se a opacificação inicial das câmaras cardíacas esquerdas.

**Figura 3:**
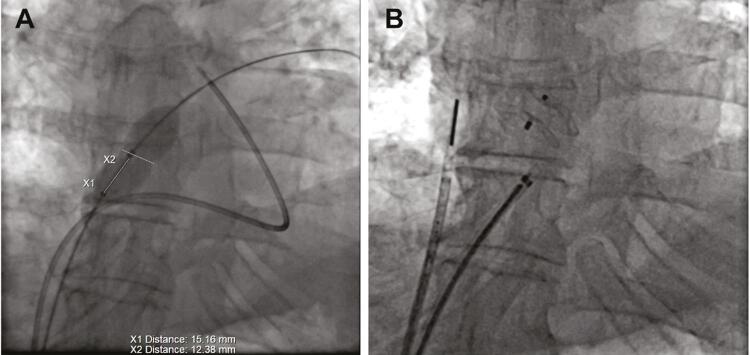
Painel A: Teste de oclusão do FOP realizado por balão dilatador no FOP; Painel B: Colocação de um dispositivo oclusivo Amplatzer® ASD de 14 mm.

No 32º dia do pós-operatório, o paciente foi submetido a cateterismo do coração direito. A pressão arterial pulmonar (PAP) estava normal (sistólica: 34 mmHg; diastólica: 9 mmHg; média: 20 mmHg). Foi realizado um teste de oclusão inflando-se um balão dilatador pelo FOP ( Figura 3A ) – a O_2_sat sistêmica aumentou de 77% para 95% e a pO_2_ arterial aumentou de 41 para 70 mmHg com ar ambiente, mantendo um PAP normal ( Tabela 1 ). A oclusão foi realizada com um dispositivo oclusivo Amplatzer® ASD de 14 mm ( Figura 3B ). No ETE de acompanhamento, não foi observado vazamento residual. Posteriormente o paciente recebeu alta em terapia antiplaquetária dupla, com uma O_2_sat de 98% em ar ambiente. O tratamento com clopidogrel foi interrompido um mês após o procedimento. Ele permaneceu assintomático no acompanhamento de um ano.

**Tabela 1:** Gasometria realizada durante cateterismo do coração direito

	Antes da oclusão do balão		Após a oclusão do balão	

	Artéria pulmonar	Artéria radial	Artéria pulmonar	Artéria radial
pH	7,46	7,47	7,41	7,41
**pCO**_**2**_ **(mmHg)**	30	26	32	29
**pO**_**2**_ **(mmHg)**	25	**41**	32	**70**
**O**_**2**_ **sat (%)**	52	**83**	65	**95**
**HCO**_**3-**_ **(mmol/L)**	21	19	20	20

## Discussão

Estima-se que a prevalência de FOP na população em geral seja de ~25%.^1^ Na maioria dos casos, o shunt interatrial é hemodinamicamente insignificante. Entretanto, em circunstâncias raras, um shunt D-E através de um FOP pode causar desoxigenação arterial clinicamente significativa misturando sangue venoso e arterial. Esses pacientes geralmente apresentam síndrome de platipneia-ortodeoxia, uma doença rara caracterizada por dispneia e desoxigenação arterial induzida pela posição em pé e geralmente aliviada quando o paciente assume a posição supino.^2^

A ocorrência de shunt interatrial D-E geralmente está associada à hipertensão pulmonar espontânea ou induzida. A ocorrência desse shunt com pressão arterial pulmonar normal é muito rara, mas já foi descrita em relatos de casos anteriores. Isso ocorre com o fluxo sanguíneo preferencial passando da veia cava inferior para o átrio esquerdo, através do FOP, mesmo na ausência de um gradiente de pressão interartrial. Uma válvula de Eustáquio proeminente e uma alteração na anatomia da câmara direita podem ser fatores que contribuem para isso. Essa síndrome foi descrita em pacientes com problemas mecânicos que causam deformidade atrial ou do septo, como a cifoescoliose,^3^ doença pulmonar restritiva, pneumonectomia prévia,^4^ efusão pleural,^5^ paralisia e ascensão diafragmática,^6^ aneurisma da aorta ascendente^3^ ou pós-toracotomia.^7^ Nesses casos, a relação anatômica entre o septo atrial e a veia cava inferior foi alterada, facilitando o redirecionamento do fluxo de sangue dessaturado através do FOP.

O histórico de sintomas pode ser curto e pode haver um aparecimento agudo, com piora rápida em alguns dias. Propriamente dito, o diagnóstico de uma síndrome de desoxigenação arterial geralmente é um diagnóstico “por exclusão”.^2^ Causas comuns de hipoxemia aguda, tais como pneumonia, insuficiência cardíaca aguda, embolia pulmonar ou outra doença pulmonar estrutural, devem ser excluídas primeiramente. No caso desse estudo, o ecocardiograma transtorácico não havia conseguido realizar o diagnóstico. A primeira dica veio da cintilografia pulmonar de V/Q solicitada para excluir a embolia pulmonar ou outros desequilíbrios de V/Q, que revelou captação do marcador pelo cérebro e pelo rim, um achado que é diagnóstico de shunt D-E.^8^ O ETE confirmou o shunt D-E pela passagem do fluxo sanguíneo da veia cava inferior para o FOP. A análise das imagens de TC revelou que a distensão abdominal devido à obstrução intestinal pós-operatória tinha causado a elevação do diafragma e a deformação cardíaca, que, neste caso, foram responsáveis pelo fluxo sanguíneo. Depois de uma extensa revisão da literatura, identificou-se que este é o primeiro caso relatado de síndrome de desoxigenação arterial devido ao FOP nessas circunstâncias. Outra característica exclusiva deste caso foi a grave hipoxemia em posição supina, ao contrário do alívio típico da desoxigenação em posição supina de pacientes com síndrome de platipneia-ortodeoxia. Isso sugere que a deformação anatômica que leva ao fluxo sanguíneo não dependia da posição corporal.

Uma possível limitação da documentação deste caso foi a não realização de uma gasometria completa em posições corporais diferentes. Isso aconteceu pelo fato de a hipoxemia grave já ter sido documentada em decúbito sem mudança significativa na oximetria de pulso em posição sentada ou em pé, e, portanto pareceu ser clinicamente desnecessário realizar mais punções da artéria radial no momento.

## Conclusão

O presente caso ilustra o diagnóstico e o tratamento bem-sucedido de uma causa rara de hipoxemia e destaca os mecanismos causadores de fluxo cardíaco anormal e oxigenação deficiente com shunts D-E, que, em casos raros, podem ocorrer apesar das pressões normais nas câmaras.
