# Supplementary material for: Uma Causa Rara de Hipoxemia após Cirurgia Ortopédica num Doente Idoso
Source: Arq Bras Cardiol. 2022 Mar 10;118(3):659–62. [Article in Portuguese] doi: 10.36660/abc.20210409 (PMC8959027; doi:10.36660/abc.20210409)
Supplement: Translation

# Translation: An Unusual Cause of Hypoxemia after Orthopedic Surgery on an Elderly Patient

**Authors:** Pedro Carvalho, Daniela Meireles, José Luís Martins, Marco Costa, Ana Briosa Neves

**Affiliations:** 1 Portugual Centro Hospitalar do Baixo Vouga – Cardiology Department, Aveiro – Portugual; 2 Portugal Centro Hospitalar do Baixo Vouga – Internal Medicine Department, Aveiro – Portugal; 3 Portugal Centro Hospitalar Universitário de Coimbra – Cardiology Department, Coimbra – Portugal

**Keywords:** Foramen Ovale, Patent, Hypoxia, Vascular Closure Devices

## Introduction

Various conditions can cause post-operative hypoxemia, especially in elderly patients. However, a new symptomatic cardiac shunt is a very rare and unexpected complication in this setting. This study presents a case of refractory hypoxemia after orthopedic surgery due to right-to-left (R-L) shunt via a patent foramen ovale (PFO).

## Case Report

A 71-year-old male underwent elective left hip replacement surgery, under loco-regional anesthesia. His medical history included obesity, hypertension, diabetes mellitus, and a stroke. He had no history of cardiopulmonary disease.

The first postoperative day was complicated by ileus ( Figure 1 ). Diet was restarted four days later, but abdominal distention and reduced bowel movements persisted. On postoperative day 15, the patient presented severe refractory hypoxemia, with an O_2_saturation (O_2_sat) of 75%, improving only to 86% on high-flow oxygen therapy (F_i_O_2_90-100%). Despite this, he was calm, showing no signs of respiratory distress. Blood pressure was 110/75 mmHg, heart rate was 76 bpm, and temperature was 36ºC. Cardiac and pulmonary auscultation were normal. There was no jugular venous distension, peripheral edema, or cyanosis.

**Figure 1:** Abdominal radiography demonstrating bowel distention.

Arterial blood gas analysis confirmed severe hypoxemia, with a pO_2_of 38 mmHg on F_i_O_2_of 28%, improving only to 49 mmHg on F_i_O_2_100%. Blood analysis was unremarkable, except for elevated d-dimers (1608 ng/mL). Electrocardiogram and bedside echocardiogram were normal. The patient underwent chest computed tomography (CT) angiography, which showed no signs of pulmonary embolism or significant parenchymal lung disease. The following days, he maintained an O_2_sat of 85-89%, despite high-flow oxygen nasal cannula, regardless of upright, supine, or left lateral decubitus body position.

A ventilation/perfusion (VQ) lung scan was performed, demonstrating the absence of VQ imbalance, but revealing a brain and kidney uptake of tracer, suggesting an R-L shunt ( Figure 2A ). Transoesophageal echocardiography (TOE) revealed an interatrial septal aneurysm and a PFO with a large resting R-L shunt visible by color Doppler and agitated saline injection ( Figure 2B ). Upon review of CT images, bowel distention was verified to have caused left hemidiaphragm elevation, changing the supra-hepatic inferior vena cava axis and heart position (and, consequently, the interatrial septal position) horizontally. ( Figure 2C ) On contrast imaging, early opacification of the left cardiac chambers was noted.

**Figure 2:** Panel A: V/Q scan demonstrating brain and kidney uptake of 99mTc-macroaggregated albumin; Panel B: TOE demonstrating PFO and interatrial septal aneurysm, with a large resting R-L shunt visible by color Doppler and agitated saline injection; Panel C: CT imaging demonstrating left hemidiaphragm elevation*, changing the supra-hepatic inferior vena cava axis* and the heart position* horizontally.

On postoperative day 32 the patient underwent right heart catheterization. Pulmonary artery pressures (PAP) were normal (systolic: 34mmHg; diastolic: 9mmHg; mean: 20mmHg). An occlusion test was performed by inflating a sizing balloon on the PFO ( Figure 3A ) - systemic O_2_sat increased from 77% to 95%, and arterial pO_2_increased from 41 to 70 mmHg in room air, while maintaining a normal PAP ( Table 1 ). Closure was performed with a 14 mm Amplatzer® ASD occluder device ( Figure 3B ). On follow-up TOE, no residual leak was noted. The patient was later discharged on dual antiplatelet therapy, with an O_2_sat of 98% in room air. Clopidogrel was stopped 1 month after the procedure. He remains asymptomatic at 1-year follow-up.

**Figure 3:** Panel A: PFO occlusion test performed by inflating sizing balloon on the PFO; Panel B: Deployment of a 14 mm Amplatzer® ASD occluder device.

**Table 1:** Blood gas analysis during right heart catheterization

	Before balloon occlusion		After balloon occlusion	

	Pulmonary artery	Radial artery	Pulmonary artery	Radial artery
pH	7.46	7.47	7.41	7.41
**pCO** ^**2**^ **(mmHg)**	30	26	32	29
**pO** ^**2**^ **(mmHg)**	25	**41**	32	**70**
**O** ^**2**^ **sat (%)**	52	**83**	65	**95**
**HCO** ^**3-**^ **(mmol/L)**	21	19	20	20

## Discussion

The prevalence of PFO in the general population is estimated to be ~25%.^1^ In most cases, the interatrial shunt is hemodynamically trivial. However, in rare circumstances, a R-L shunt through a PFO may cause clinically significant arterial deoxygenation by mixing venous and arterial blood. These patients usually present a platypnea-orthodeoxia syndrome, a rare condition characterized by dyspnea and arterial deoxygenation induced by an upright position and typically relieved by lying supine.^2^

The occurrence of R-L interatrial shunting is usually associated with spontaneous or induced pulmonary hypertension. The occurrence of this shunt with normal pulmonary artery pressure is very uncommon, but has been described in previous case reports. This occurs by preferential blood flow streaming from the inferior vena cava into the left atrium, through the PFO, even in the absence of an interatrial pressure gradient. A prominent Eustachian valve and right chamber anatomy modification can act as contributing factors. Such a syndrome has been described in patients with mechanical conditions, causing atrial or septal deformity, such as kyphoscoliosis,^3^ restrictive lung disease, previous pneumonectomy,^4^ pleural effusion,^5^ diaphragmatic paralysis and ascension,^6^ ascending aorta aneurysm,^3^ or post-thoracotomy.^7^ In these cases, the anatomic relationship between the atrial septum and the inferior vena cava was changed, facilitating desaturated blood flow redirection through the PFO.

The history of symptoms can be short and may have an acute onset with rapid worsening within a few days. As such, the diagnosis of an arterial deoxygenation syndrome is usually a “rule-out” diagnosis.^2^ Common causes of acute hypoxemia must be first excluded, such as pneumonia, acute heart failure, pulmonary embolism, or other structural lung disease. In our case, transthoracic echocardiography had missed the diagnosis. The first hint came from the VQ lung scan, requested to rule out pulmonary embolism or other VQ imbalances, which revealed kidney and brain uptake of tracer, a finding that is diagnostic of a R-L shunt.^8^ TOE confirmed the R-L shunt through the streaming of blood flow from the inferior vena cava to the PFO. Upon review of CT images, we found that the abdominal distention due to postoperative ileus had caused diaphragm elevation and cardiac deformation, which in this case was responsible for the blood streaming. After an extensive review of the literature, we found that this is the first reported case of an arterial deoxygenation syndrome due to PFO under these circumstances. Another unique feature of this case was the severe hypoxemia while lying supine, as opposed to the typical relief of deoxygenation in the supine position of patients with platypnea-orthodeoxia syndrome. This suggests that the anatomic deformation leading to blood streaming was independent of the body position.

A potential limitation of the documentation of this case was that a thorough blood gas analysis in different body positions was not performed. This was due to the fact that severe hypoxemia had already been documented in decubitus, with no significant change in pulse oximetry in the sitting or standing position, so additional radial puncture seemed clinically futile at the time.

## Conclusion

The present case illustrates the diagnosis and successful treatment of a rare cause of hypoxemia and highlights the mechanisms causing abnormal cardiac flow and impaired oxygenation with cardiac R-L shunts, which in rare cases can occur despite normal chamber pressures.
